# Postload Glycated Albumin as an Alternate Measure for Diabetes Screening in a Chinese Population

**DOI:** 10.1155/2018/7932528

**Published:** 2018-05-22

**Authors:** Hang Su, Junling Tang, Xiaojing Ma, Xingxing He, Lingwen Ying, Yufei Wang, Yuqian Bao, Jian Zhou

**Affiliations:** Department of Endocrinology and Metabolism, Shanghai Clinical Center for Diabetes, Shanghai Diabetes Institute, Shanghai Key Laboratory of Diabetes Mellitus, Shanghai Jiao Tong University Affiliated Sixth People's Hospital, Shanghai 200233, China

## Abstract

In previous epidemiological screening in China, glycated albumin (GA) was mostly detected during the fasting state. This strict restriction causes some problems with diabetes screening. It is unclear if GA could help improve the efficiency of screening for diabetes for subjects who are not in the fasting state. The present study analyzed the differences between fasting and postload (30, 60, 120, and 180 min) GA levels. A total of 691 participants were enrolled in the present study. The Bland-Altman difference plots revealed that 95.4, 94.8, 93.6, and 93.9% of data points were within the limits of agreement for each time point. The receiver operating characteristic curve showed that the areas under the curve (AUC) for baseline GA and postload GA for every time point were 0.822 (95% CI 0.791–0.849), 0.821 (95% CI 0.790–0.848), 0.833 (95% CI 0.803–0.860), 0.840 (95% CI 0.811–0.867), and 0.840 (95% CI 0.810–0.867), with sensitivities of 67.5, 68.1, 69.3, 71.6, and 69.3%, respectively. There was no difference between the baseline and postload GA levels in either AUC or sensitivity (all *p* > 0.05). In conclusion, postload serum GA levels were in good agreement with those at baseline, and thus, it may be reasonable to employ nonfasting measurements of GA levels for diabetes screening.

## 1. Introduction

For clinical monitoring indicators, the value of a nonfasting measurement is an important determinant of its extended application in the clinical setting. For example, in recent years, nonfasting lipid profile measurements have been vigorously promoted by numerous studies [1]. Similarly, the levels of glycated hemoglobin A_1c_ (HbA_1c_), a standard blood glucose monitoring indicator, have been shown to remain stable after a glucose load in a previous study [2]. It is expected that increased flexibility regarding blood sampling time could improve patient adherence.

As an emerging indicator, glycated albumin (GA) has been proposed to be an effective supplement to HbA_1c_ for blood glucose monitoring. Recently, many studies have suggested that GA may be an effective marker for screening for diabetes mellitus (DM) [3–5]. We previously reported that GA was a sensitive and specific indicator for diabetes screening in Chinese subjects [5]. In previous epidemiological screening in China, GA was mostly detected during the fasting state. This strict restriction causes some problems with diabetes screening. It is unclear if GA could help improve the efficiency of screening for diabetes for subjects who are not in the fasting state.

However, few studies have explored the changes in serum GA levels after a glucose load, especially in the Chinese population. Nevertheless, we have no information on the value of postload GA for diabetes screening and diagnosis. Published data include only small sample-sized studies that have used relatively simple evaluations and drawn inconsistent conclusions. Some studies found no significant changes in serum GA levels after a glucose load [6, 7], whereas Hashimoto et al. [8] showed that the 2-hour postprandial GA levels were slightly increased in diabetes patients with poor glycemic control. Therefore, to provide evidence for clinical application of GA measurements, this study aimed at comparing results between fasting and nonfasting GA levels and providing an analysis of diabetes screening efficiency for postload GA in a large population sample with different glucose tolerance statuses.

## 2. Materials and Methods

### 2.1. Study Population

The study population comprised of 691 participants, including 178 with normal glucose tolerance, 178 with impaired glucose regulation, and 335 who were newly diagnosed with antihyperglycemic agent-naive DM, who presented in the clinic of the Department of Endocrinology and Metabolism of Shanghai Jiao Tong University Affiliated Sixth People's Hospital from January 2014 to January 2016. The population was restricted to those without a history of thyroid dysfunction, chronic liver disease, nephrotic syndrome, hypoalbuminemia, tumors, mental disorders, acute infection, pregnancy, or glucocorticoids therapy.

This study was approved by the Ethics Committee of Shanghai Jiao Tong University Affiliated Sixth People's Hospital. Written informed consents were provided by all participants prior to enrollment.

### 2.2. Physical and Laboratory Assessments

All participants completed a standard questionnaire in the outpatient department after overnight (8–10 hours) fasting. The questionnaire collected details of the patients' medical histories, including the patients' histories of past and present illnesses, medication histories, and family histories. Physical examination included measurements of height, weight, and blood pressure. The body mass index (BMI) was calculated as weight/height^2^ (kg/m^2^).

Blood samples were collected to measure the fasting levels of plasma glucose (PG_0_), serum GA (GA_0_), and HbA_1c_. Postload levels of plasma glucose (PG_30_, PG_60_, PG_120_, and PG_180_) and serum GA (GA_30_, GA_60_, GA_120_, and GA_180_) were measured at 30, 60, 120, and 180 min after administration of 75 g of oral glucose (oral glucose tolerance test, OGTT). The plasma glucose levels were immediately obtained via the glucose oxidase method (Kehua Biological Engineering Co., Ltd., Shanghai, China) using the Glamour 2000 biochemical autoanalyzer. GA levels were measured via an enzymatic method using an enzyme-based assay kit (Lucica GA-L, Asahi Kasei Pharma, Tokyo, Japan) on a 7600–120 autoanalyzer (Hitachi, Tokyo, Japan) with intra- and interassay coefficients of variation (CVs) of 1.47–3.30% and 1.95–4.73%, respectively. HbA_1c_ levels were detected by high-performance liquid chromatography (HPLC, Variant II hemoglobin analyzer; Bio-Rad, Hercules, CA, USA) with intra- and interassay CVs of 0.55–2.58% and 0.75–3.39%, respectively.

### 2.3. Diagnostic Criteria

DM and impaired glucose regulation were diagnosed according to the 1999 World Health Organization criteria [9].

### 2.4. Consistency Analysis

A mountain plot was created by computing a percentile rank for ranked differences between paired postload and baseline GA levels and cumulative percentages (*y*-axis values) against the ranked differences (*x*-axis values) to evaluate the agreement between postload and baseline GA levels [10]. The Bland-Altman difference plot was used to depict the differences between the paired postload and baseline GA levels after log transformation (baseline logGA minus postload logGA result along the *y*-axis against the average of the baseline logGA and postload logGA along the *x*-axis) [11]. The 95% confidence intervals (CIs) for the difference ranges (the sample mean difference ± 1.96 standard deviation) reflected the 95% probability range in which the mean difference population parameter lies [12]. If more than 95% of data points fell within these limits of agreement, there was not a significant systematic difference between the two points of time for the measurement.

### 2.5. Statistical Analysis

All statistical analyses were performed using SPSS 19.0 and MedCalc statistical software version 15.2. Data are presented as the means ± standard deviation. Each variable was examined for a normal distribution, and pair analyses were carried out using a paired Student's *t*-test and Wilcoxon signed-rank sum test. The chi-square test was used for intergroup comparisons of categorical variables. Intergroup comparisons of skewed data were made using the Kruskal–Wallis test. The absolute relative errors (AREs) were calculated to assess the differences in the postload and baseline GA levels. Spearman correlation analysis was performed to explore the agreement in the postload GA levels. The receiver operating characteristic (ROC) curve was plotted to assess the power of the postload GA as a screening test to discriminate diabetes patients from nondiabetes patients. The mountain plot and Bland-Altman difference plots were used to identify the bias in the postload GA levels. A two-tailed *p* value of <0.05 was considered to be statistically significant.

## 3. Results

### 3.1. Clinical Characteristics of the Study Participants

The study included a total of 691 participants of 323 men and 368 women, including 356 without DM (non-DM group) and 335 with DM. Compared to the non-DM participants, patients in the DM group were older and had significantly higher BMI, systolic blood pressure, diastolic blood pressure, and HbA_1c_, and PG and GA levels at all measurement time points (all *p* < 0.001, Table 1).

### 3.2. Analysis of Agreement for the Postload and Baseline GA Levels

**Figure 1:**
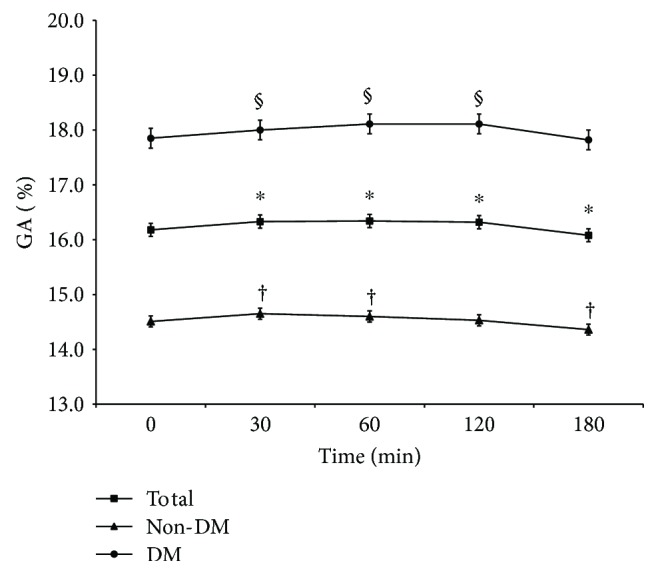
Serum GA levels for the entire study population, the DM group, and the non-DM group. Data are presented as the means ± standard deviation. ^∗^*p* < 0.01 versus fasting GA levels in the entire study population; ^†^*p* < 0.01 versus fasting GA levels in the non-DM group; ^§^*p* < 0.01 versus fasting GA levels in the DM group.

**Figure 2:**
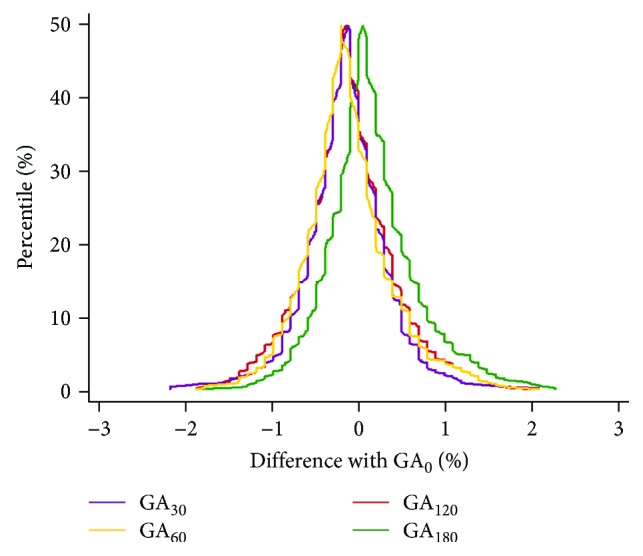
Mountain plot of agreement between postload and baseline GA levels in all participants (cumulative percentages with a lower or equal value to the score under consideration along the *y*-axis against the postload GA levels minus baseline GA levels along the *x*-axis).

**Figure 3:**
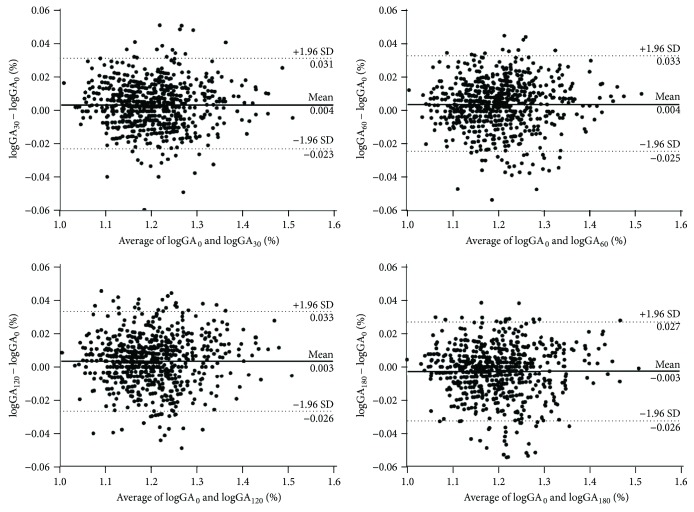
Bland-Altman difference plots showing the differences between baseline and postload GA levels after log transformation for all participants (baseline logGA minus postload logGA result along the *y*-axis against the average of the baseline logGA and postload logGA along the *x*-axis). Horizontal lines are drawn at the mean difference (solid line) and the limits of agreement (both upper and lower, dashed lines).

The obtained measurements demonstrated that GA levels at 30 and 60 min postload were slightly elevated compared with the baseline levels in both the non-DM and DM groups (all *p* < 0.01, Figure 1). Spearman correlation analysis revealed that baseline GA levels were positively associated with postload GA levels at every time point for all participants (*r* = 0.977 to 0.981, all *p* < 0.01). The AREs for postload GA levels at 30, 60, 120, and 180 min were 2.6 ± 2.1%, 2.8 ± 2.2%, 2.8 ± 2.2%, and 2.6 ± 2.3%, respectively. The mountain plot showed that the mountain peaked at approximately *x* = 0 and was symmetric around the line of *x* = 0 without significant shifts. Most of the differences were within ±1 standard deviation, indicating high agreement between the postload and baseline GA levels (Figure 2). The Bland-Altman difference plots revealed that the mean differences and 95% CIs between the postload GA levels at every time point and baseline GA measurements after log transformation were 0.004% (−0.023–0.031%), 0.004% (−0.025–0.033%), 0.003% (−0.026–0.033%), and−0.003% (−0.032–0.027%), respectively. On this graph, 95.4, 94.8, 93.6, and 93.9% of the data points for GA_30_, GA_60_, GA_120_, and GA_180_ fell within the limits of agreement, respectively (Figure 3).

### 3.3. Screening of Efficiency of Postload and Baseline GA for Diabetes

**Figure 4:**
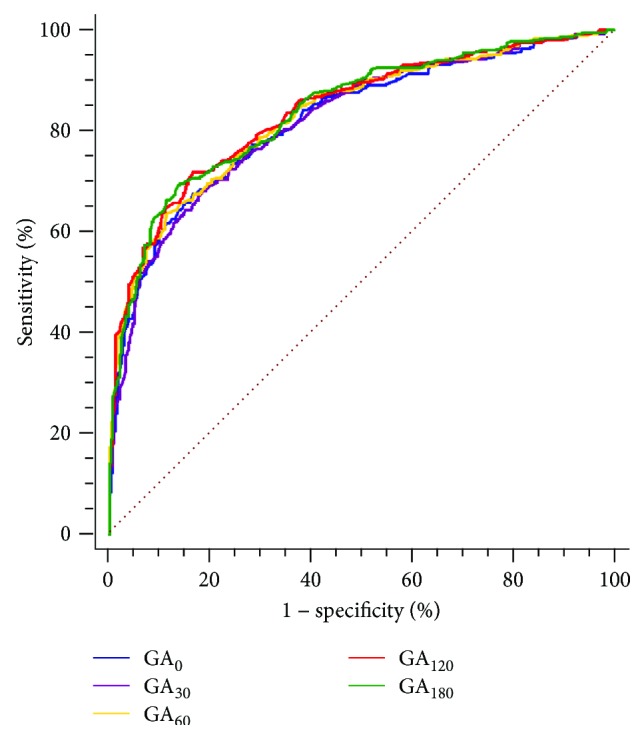
ROC curves of postload and baseline GA for screening diabetes.

The ROC curve was plotted to examine the predictive values of the postload and baseline GA levels for identifying undiagnosed diabetes. The optimal cut-off point of the baseline GA was 16.3% with a sensitivity, specificity, positive predictive value, and negative predictive value of 67.5% (95% CI: 62.2–72.5%), 83.4% (95% CI: 79.2–87.1%), 79.3% (95% CI: 74.1–83.9%), and 73.2% (95% CI: 68.6–77.4%), respectively. The diagnostic indices for screening diabetes with postload GA are shown in Table 2. The McNemar test revealed that the postload GA levels at every time point exhibited an equivalent sensitivity as baseline GA for identifying diabetes (all *p* > 0.05). The areas under the curve (AUC) for baseline GA and postload GA at every time point were 0.822 (95% CI 0.791–0.849), 0.821 (95% CI 0.790–0.848), 0.833 (95% CI 0.803–0.860), 0.840 (95% CI 0.811–0.867), and 0.840 (95% CI 0.810–0.867), respectively. There were no differences in the AUC for the baseline GA levels and the postload GA levels at every time point (*p* = 0.965, 0.620, 0.413, and 0.413, Figure 4).

## 4. Discussion

The current study provides the first evaluation of the screening efficiency of serum GA levels after glucose load in a Chinese population with differing glucose tolerance statuses. The results demonstrated that the AREs for postload GA levels at all measurement time points (30, 60, 120, and 180 min postload) were all within 5%, and more than 93.6% of data points from each measurement time point were found to be within the limits of agreement of the baseline level. There was no difference between the sensitivity of the baseline versus postload GA for screening diabetes. Moreover, the ROC curve showed equal levels of baseline and postload GA for screening diabetes, which indicates that postload GA can also guide diabetes screening.

Serum GA can reflect short-term (2–3 weeks) mean glycemic levels [13–18], and the measurement of GA levels has distinctive advantages in patients with newly diagnosed DM, marked glycemic excursion, treatment adjustment, and stress [19]. Recent studies have focused on the role of GA as a direct pathological harmful factor in the development of vascular complications in DM [14, 20]. Additionally, GA cannot only predict the development but also indicate the severity of DM complications [21–24]. Recently, many studies have assessed the value of GA as an effective marker for DM screening [3–5, 25]. Our recently study also showed that the additional measurement of GA could help prevent the misdiagnosis of diabetes [26], which is consistent with the findings of the present study. The different population constituents may explain the cut-off inconsistency. In addition, we used the ROC curve for further verification. The result suggested that nonfasting GA could also help indirect diabetes screening, which improves the efficiency and convenience of assessing diabetes.

To date, few studies have assessed the agreement between postload and baseline GA levels. Shima et al. [6] investigated the diurnal variation in GA levels in 15 DM patients via HPLC analysis and reported almost no change in the GA levels. A study in Taiwan involving 12 individuals without DM who underwent 75 g of OGTT showed via pair analysis that the GA levels were similar in fasting and postprandial samples [7]. However, Hashimoto et al. [8] found that the GA levels increased between preprandial and 2 h postprandial specimens in 16 Japanese DM patients with poor glycemic control (*p* < 0.05), especially after breakfast (*p* < 0.05) and observed a significant correlation between the variation ranges of GA and blood glucose measurements (*r* = 0.322, *p* = 0.021). The present study utilized the Wilcoxon signed-rank sum test and Spearman correlation to evaluate the central tendency and correlation, respectively. The discrete tendency was evaluated by generating a mountain plot and Bland-Altman difference plots. We confirmed that although there was a slight increase, postload GA levels were in good agreement with baseline GA levels. We further investigated the screening efficiency of postload GA in our study population of 691 Chinese individuals with varying glucose metabolism statuses. The results indicated that it is reasonable to employ nonfasting measurements of GA levels for diabetes screening.

These inconsistencies in the variation of GA after glucose load may be attributed to different factors, such as variations in blood sampling, storage processes, and methodological assessment. The mechanisms responsible for the minor changes in GA levels after application of a glucose load remain unclear and may be related to different glycated reactions of GA and HbA_1c_. In HbA_1c_, valine is glycated, whereas lysine is glycated in GA. This difference in the glycated amino acid may lead to the different kinetics of the early Amadori reaction. Day et al. [27] reported that the glycation reaction of GA progressed approximately 10 times faster than that of HbA_1c_ in a Sprague–Dawley rat model of DM. This is believed to be because when the elevated blood glucose level decreases, an unstable product of early HbA_1c_ through the Amadori reaction is reversibly dissociated to hemoglobin and glucose [28], whereas unstable GA produced from albumin and glucose rapidly goes through the irreversible reaction, and stable GA is produced [14]. Further studies are needed to fully elucidate the underlying mechanisms.

There are some limitations in this study. First, this was a single-center study, and data from multiple centers are needed to further confirm the findings. Second, serum GA levels were evaluated at only four time points after the glucose load. Data from more measurement time points could provide a more complete picture of the GA variation after the glucose load.

## 5. Conclusion

This study showed that postload GA levels were in good agreement with baseline GA levels in Chinese individuals with different glucose tolerance statuses. These results support that the nonfasting GA measurement has improved convenience and equal effectiveness for diabetes assessments. Thus, the nonfasting GA measurement may contribute to greater patient adherence to diabetes screening. Finally, this study provides a foundation for further application of GA measurements for diabetes screening.

## Figures and Tables

**Table 1:** Demographic and clinical characteristics of study participants.

Variable	Total (*n* = 691)	Non-DM (*n* = 356)	DM (*n* = 335)	*p* value
*n* (men/women)	323/368	141/215	182/153	<0.001
Age (years)	50.5 ± 13.3	46.7 ± 14.0	53.6 ± 11.7	<0.001
BMI (kg/m^2^)	24.7 ± 3.3	24.2 ± 3.3	25.3 ± 3.2	<0.001
Systolic blood pressure (mmHg)	130.1 ± 17.9	127.1 ± 17.3	135.3 ± 17.6	<0.001
Diastolic blood pressure (mmHg)	79.4 ± 10.8	77.0 ± 10.5	82.0 ± 10.6	<0.001
HbA_1c_ (%)	6.1 ± 0.9	5.6 ± 0.4	6.7 ± 1.0	<0.001
HbA_1c_ (mmol/mol)	43 ± 10	38 ± 5	49 ± 11	<0.001
PG_0_ (mmol/L)	6.6 ± 1.5	5.7 ± 0.6	7.7 ± 1.5	<0.001
PG_30_ (mmol/L)	11.2 ± 2.6	9.7 ± 1.7	12.8 ± 2.3	<0.001
PG_60_ (mmol/L)	12.9 ± 4.1	10.1 ± 2.7	15.9 ± 3.0	<0.001
PG_120_ (mmol/L)	11.0 ± 4.8	7.4 ± 1.8	14.9 ± 3.9	<0.001
PG_180_ (mmol/L)	7.5 ± 3.8	5.2 ± 1.6	9.9 ± 3.9	<0.001
GA_0_ (%)	16.2 ± 3.1	14.6 ± 1.9	17.8 ± 3.3	<0.001
GA_30_ (%)	16.3 ± 3.2	14.7 ± 2.0	18.0 ± 3.3	<0.001
GA_60_ (%)	16.3 ± 3.2	14.7 ± 1.9	18.1 ± 3.3	<0.001
GA_120_ (%)	16.3 ± 3.2	14.6 ± 1.8	18.1 ± 3.4	<0.001
GA_180_ (%)	16.1 ± 3.1	14.4 ± 1.8	17.8 ± 3.3	<0.001

Data are presented as mean ± standard. BMI: body mass index; HbA_1c_: glycated hemoglobin A_1c_; PG_0_: fasting plasma glucose; PG_30_: 30 min postload plasma glucose; PG_60_: 60 min postload plasma glucose; PG_120_: 120 min postload plasma glucose; PG_180_: 180 min postload plasma glucose; GA_0_: fasting glycated albumin; GA_30_: 30 min glycated albumin; GA_60_: 60 min glycated albumin; GA_120_: 120 min glycated albumin; GA_180_: 180 min glycated albumin.

**Table 2:** Diagnostic indices for screening diabetes with postload and baseline GA.

Criteria	Sensitivity (%)	Specificity (%)	Positive predictive value (%)	Negative predictive value (%)
GA_0_ ≥ 16.3%	67.5 (95% CI: 62.2–72.5%)	83.4% (95% CI: 79.2–87.1%)	79.3 (95% CI: 74.1–83.9%)	73.2% (95% CI: 68.6–77.4%)
GA_30_ ≥ 16.4%	68.1 (95% CI: 62.8–73.0%)	81.5% (95% CI: 77.0–85.4%)	77.6 (95% CI: 72.4–82.2%)	73.1% (95% CI: 68.4–77.4%)
GA_60_ ≥ 16.4%	69.3 (95% CI: 64.0–74.2%)	80.6% (95% CI: 76.1–84.6%)	77.1 (95% CI: 71.9–81.7%)	73.6% (95% CI: 68.9–77.9%)
GA_120_ ≥ 16.3%	71.6 (95% CI: 66.5–76.4%)	83.4% (95% CI: 79.2–87.1%)	80.3 (95% CI: 75.3–84.6%)	75.8% (95% CI: 71.2–79.9%)
GA_180_ ≥ 16.2%	69.3 (95% CI: 64.0–74.2%)	86.0% (95% CI: 82.2–89.6%)	82.6 (95% CI: 77.6–86.8%)	74.9% (95% CI: 70.4–79.0%)

GA: glycated albumin; CI: confidence interval.

## Abbreviations

ARE: Absolute relative error
AUC: Area under the curve
BMI: Body mass index
CI: Confidence interval
CV: Coefficients of variation
DM: Diabetes mellitus
GA: Glycated albumin
HbA_1c_: Glycated hemoglobin A_1c_
HPLC: High-performance liquid chromatography
OGTT: Oral glucose tolerance test
PG: Plasma glucose
ROC: Receiver operating characteristic.
